# High Genetic Differentiation between the M and S Molecular Forms of *Anopheles gambiae* in Africa

**DOI:** 10.1371/journal.pone.0001968

**Published:** 2008-04-16

**Authors:** Caroline Esnault, Matthieu Boulesteix, Jean Bernard Duchemin, Alphonsine A. Koffi, Fabrice Chandre, Roch Dabiré, Vincent Robert, Frédéric Simard, Frédéric Tripet, Martin J. Donnelly, Didier Fontenille, Christian Biémont

**Affiliations:** 1 Laboratoire de Biométrie et Biologie Evolutive (UMR 5558), CNRS, Université de Lyon, Université Lyon1, Villeurbanne, France; 2 Centre de Recherche Médicale et Sanitaire (CERMES), Réseau International de l'Institut Pasteur, Niamey, Niger; 3 Institut Pierre Richet, Institut National de Santé Publique, Abidjan, Côte d'Ivoire; 4 Unité de Recherche 016, Institut de Recherche pour le Développement (IRD), CREC, Cotonou, Bénin; 5 Institut de Recherche en Sciences de la Santé (IRSS), Bobo Dioulasso, Burkina Faso; 6 Unité de Recherche 77, Institut de Recherche pour le Développement (IRD), Unité Scientifique du Muséum 504, Muséum National d'Histoire Naturelle, Paris, France; 7 Institut Pasteur, Antananarivo, Madagascar; 8 Laboratoire de Recherche sur le Paludisme, Organisation de Coordination pour la lutte contre les Endémies en Afrique Centrale (OCEAC), Yaoundé, Cameroun; 9 Unité de Recherche 016, Institut de Recherche pour le Développement (IRD), Montpellier, France; 10 Centre for Applied Entomology and Parasitology, School of Life Sciences, Keele University, Staffordshire, United Kingdom; 11 Vector Group, Liverpool School of Tropical Medicine, Pembroke Place, Liverpool, United Kingdom; University of Montreal, Canada

## Abstract

**Background:**

*Anopheles gambiae*, a major vector of malaria, is widely distributed throughout sub-Saharan Africa. In an attempt to eliminate infective mosquitoes, researchers are trying to develop transgenic strains that are refractory to the *Plasmodium* parasite. Before any release of transgenic mosquitoes can be envisaged, we need an accurate picture of the differentiation between the two molecular forms of *An. gambiae*, termed M and S, which are of uncertain taxonomic status.

**Methodology/Principal Findings:**

Insertion patterns of three transposable elements (TEs) were determined in populations from Benin, Burkina Faso, Cameroon, Ghana, Ivory Coast, Madagascar, Mali, Mozambique, Niger, and Tanzania, using Transposon Display, a TE-anchored strategy based on Amplified Fragment Length Polymorphism. The results reveal a clear differentiation between the M and S forms, whatever their geographical origin, suggesting an incipient speciation process.

**Conclusions/Significance:**

Any attempt to control the transmission of malaria by *An. gambiae* using either conventional or novel technologies must take the M/S genetic differentiation into account. In addition, we localized three TE insertion sites that were present either in every individual or at a high frequency in the M molecular form. These sites were found to be located outside the chromosomal regions that are suspected of involvement in the speciation event between the two forms. This suggests that these chromosomal regions are either larger than previously thought, or there are additional differentiated genomic regions interspersed with undifferentiated regions.

## Introduction

Malaria causes the deaths of more than one million people each year, mostly in Africa (WHO/UNICEF World Malaria Report 2005). This disease and the relevant mortality are due to one of four *Plasmodium* species, which are transmitted by mosquitoes. *Anopheles gambiae* is the major vector in sub-Saharan Africa, which has the greatest disease burden. Various methods have been developed to control mosquitoes. However, the failure of traditional measures together with the spread of insecticide- resistance in natural vector populations [1], [2], have spurred on attempts to find alternative, unconventional approaches. One of the most innovative strategies sets out to replace the entire wild populations of *An. gambiae* with genetically modified, *Plasmodium*-resistant individuals. This idea seems more plausible following the successful genetic transformation of some anopheline species, including *An. gambiae*, and the identification of putative target genes and gene drive mechanisms [3]. To ensure that the transgene spreads throughout the entire wild populations, however, we need to understand the population structure and level of gene flow of mosquito populations. This makes it very important to know whether the genetic differentiation between the two ‘molecular forms’ of *An. gambiae*, termed M and S, which are suspected of currently undergoing speciation [4]–[6], is a general phenomenon affecting all African populations. The distinction between the two forms was primarily based on sequence polymorphism in ribosomal DNA loci [7], which was subsequently confirmed by microsatellite data in Cameroon [8] and Mali [9], by the insertion patterns of various transposable elements (TEs) in Cameroon [10] and of the short interspersed nuclear elements (SINEs) *Maque* and *SINE200* in Burkina Faso, Central African Republic, Mali, and Kenya [11], [12]. The *kdr* allele, which confers knock-down resistance to pyrethroid insecticides and dichlorodiphenyltrichloroethane (DDT), was mainly present in S individuals, and so this too was segregated between the two molecular forms [see 4], [12 for reviews]. Studies of the gene flow within and between the two molecular forms revealed, however, complex patterns of differentiation. Some analyses revealed greater differences between ecological zones [13], and between allopatric populations of a given molecular form than between the M and S populations [14], suggesting that the M and S speciation is not yet complete. Some data suggest that islands of speciation are present within the genomes of these two forms, mostly in the region near the centromeres of the X and 2L chromosomes and in a region of the 2R chromosome, whereas genetic differentiation remains weak in other regions of the genome [15]–[18]. This could explain why the estimates of genetic differentiation between the M and S forms vary depending on the type of markers used, and the location of the markers in the genome [8], [9], [13], [19]. To find out whether the genetic differentiation between the M and S molecular forms is found throughout the geographical range of *An. gambiae*, we studied the insertion polymorphism of three TEs. Because the insertion sites of these TEs were scattered throughout the *An. gambiae* genome, this study provides an overview of a large portion of the mosquito genome. The insertion patterns of these TEs reveal clear differentiation between the M and S forms, whatever the geographical origin of the populations.

## Results and Discussion

**Figure 1:**
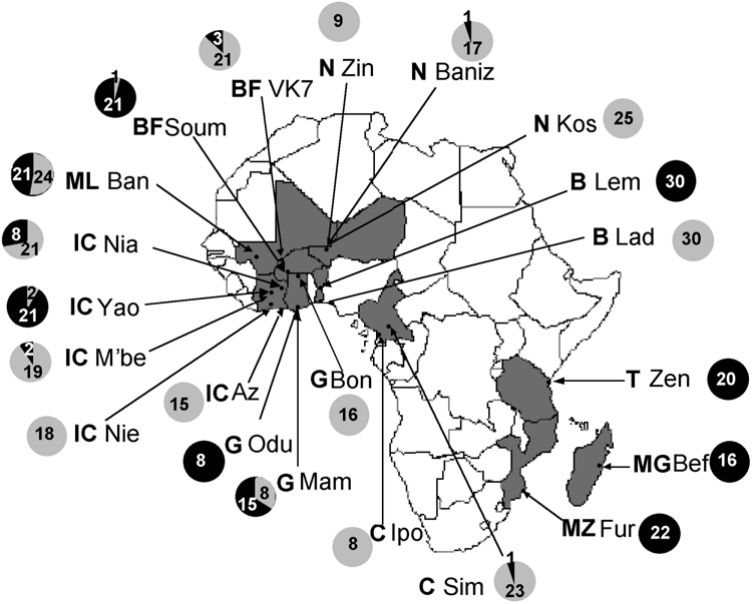
Geographic origin of the African *An. gambiae* populations. Sample sizes of the M and S molecular forms in each population are indicated in gray and black, respectively. Az = Azureti, Ban = Bankoumana, Baniz = Banizoumbou, Bef = Beforona, Bon = Bonia, Fur = Furvela, Ipo = Ipono, Kos = Kosseye, Lad = Ladji, Lem = Lema, Mam = Mampong, M'be = M'be, Nia = Niamoue, Nie = Nieky, Odu = Odumasy, Sim = Simbok, Soum = Soum, VK7 = Vallée du Kou, Yao = Yaokoffikro, Zen = Zenet, Zin = Zindarou. The populations were from B = Benin, BF = Burkina Faso, C = Cameroon, G = Ghana, IC = Ivory Coast, MG = Madagascar, ML = Mali, MZ = Mozambique, N = Niger, T = Tanzania.

**Figure 2:**
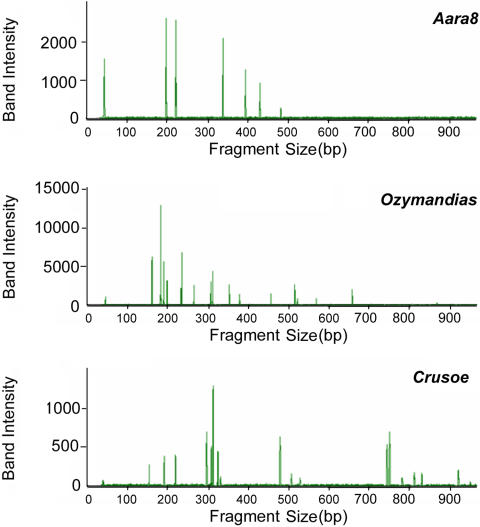
Example of individual TE profiles obtained by Transposon Display with *Aara8*, *Ozymandias*, and *Crusoe*. Each peak in the TE profile corresponds to one TE insertion in the individual analyzed. Because the probability that two TE copies would be inserted independently at the same site in two different individuals is negligible, fragments of the same size were assumed to be of identical descent, and each TE fragment of a given size was considered to be a single insertion. The matrices of the presence/absence of peaks were used to estimate genetic distances and do the PCoA analyses shown in Fig. 3, 4, and 5.

**Figure 3:**
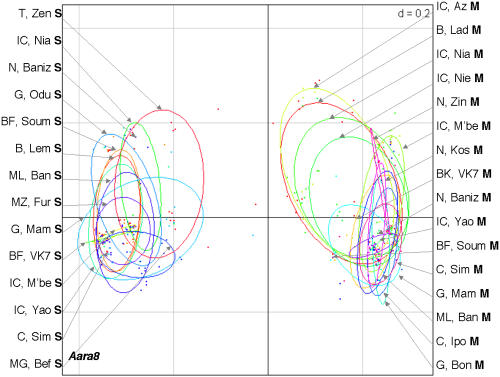
Principal Coordinate Analyses (PCoA) of the *Aara8* profiles obtained by the Transposon Display technique. The ellipses, calculated for each population, were centered on the gravity centre of each scatterplot, and the size of their axes was equal to 1.5 (the square root of the eigen values of the covariance matrix) times the standard deviation of the coordinates of the projections on the first and second axes. The percentage of variance explained by the first two axes was 75.4%, and the F value of MANOVA test between individuals of each molecular form was equal to 18.6 (p<0.001).

Twenty-one *An. gambiae* populations from ten African countries were studied: two populations from Benin, two from Burkina Faso, two from Cameroon, three from Ghana, five from Ivory Coast, one from Madagascar, one from Mali, one from Mozambique, three from Niger, and one from Tanzania (see Fig. 1, which also indicates the number of mosquitoes of the M and S molecular forms in each population). The M and S forms were distinguished on the basis of their rDNA sequence polymorphism. The non-Long Terminal Repeat (LTR) retrotransposon *Aara8*, the LTR retrotransposon *Ozymandias*, and the DNA transposon *Crusoe* were taken into consideration [10]. Individual TE insertion profiles were obtained by the Transposon Display method [20], [21]. This technique is very similar to the Sequence-Specific Amplification Polymorphism, except that the PCR amplifies a DNA sequence defined by one primer anchoring to a conserved region of the TE, and another primer anchoring to an adaptor attached to flanking sites generated by enzymatic restriction digestion. The presence and absence of TE insertions can thus be scored in individuals (Fig. 2). We therefore compared the TE insertion profiles of individuals from all the populations by estimating the inter-population differentiation indices, Φst. This parameter is analogous to Fst, and can be used to analyze the presence/absence data [22] obtained by Transposon Display. The mean Φst values between the M and S populations (0.57±0.07 for *Aara8*, 0.19±0.04 for *Ozymandias*, 0.23±0.05 for *Crusoe*), are higher than the Φst between M populations (0.12±0.06, 0.07±0.04, and 0.11±0.07, for *Aara8*, *Ozymandias*, and *Crusoe*, respectively) or S populations (0.10±0.06, 0.12±0.09, and 0.12±0.07, for *Aara8*, *Ozymandias*, and *Crusoe*, respectively) (see Table S1 for the between-population Φst and the associated P-values). A graphical representation using a Principal Coordinate Analysis (PCoA) (Fig. 3–5) clearly distinguishes between individuals of these two forms, whatever the TE and the population considered, and shows that individuals of a given molecular form cluster together. This similarity of the results for all three TEs is reinforced by significant Pearson correlation coefficient values between the Φst values obtained for the three TEs (r = 0.72 for *Aara8 vs Ozymandias*; r = 0.70 for *Aara8 vs Crusoe*; r = 0.77 for *Ozymandias vs Crusoe*; P-values<1×10^−4^). These data thus clearly reveal a high degree of differentiation between the M and S molecular forms in all 21 populations studied, with some “specific” TE insertion sites being present at high frequency in one or other form (Table 1). Among 20 such sites, 6 were found in all individuals (4 on M and 2 on S), whereas 14 were present at high frequency in one form or the other (see Fig 6). To check for possible differentiation between populations of the M or S forms, we did Principal Coordinate Analysis (PCoA) on either the M or the S populations. No structuration between populations of either the M or S form was detected, which suggests the absence of specific insertion sites for groups of populations apart from the M and S forms. This indicates that most insertion sites were widespread and highly polymorphic between populations.

**Figure 4 pone-0001968-g004:**
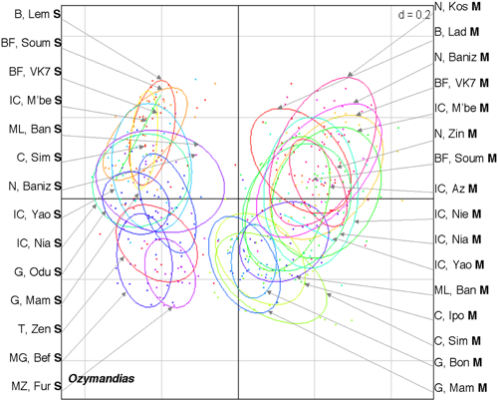
Principal Coordinate Analyses (PCoA) of the *Ozymandias* profiles. The percentage of variance explained by the first two axes was 55.1%, and the F value of MANOVA test between individuals of each molecular form was equal to 4.3 (p<0.001).

**Figure 5:**
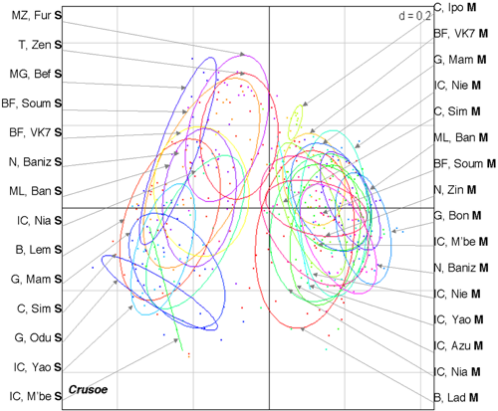
Principal Coordinate Analyses (PCoA) of the *Crusoe* profiles. The percentage of variance explained by the first two axes was equal to 55.0% and the F value of MANOVA test between individuals of each molecular form was equal to 4.1 (p<0.001).

**Figure 6:**
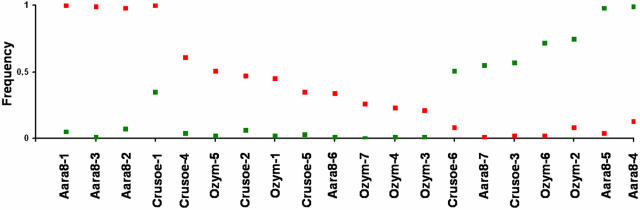
Frequencies of the 20 insertion sites sequenced in the M (red squares), and S (green squares) molecular forms. Insertion sites localized on the chromosomes: *Crusoe*-1-2, *Ozym*-1. Insertion sites localized on the unknown chromosome: *Aara8*-2-3-5. Insertion sites integrated within other transposable elements or repeated sequences: *Aara8*-1-4-6, *Ozym*-2-5, *Crusoe*-3-4. Insertion sites not isolated: *Aara8*-7, *Ozym*-3-4-6-7, *Crusoe*-5-6.

**Table 1:** Number of insertion sites and average site numbers±SE of *Aara8*, *Ozymandias*, and *Crusoe* for the populations of the S and M molecular forms.

	Number of insertion sites				Mean insertion sites number±SE	
	Specific to M molecular form	Specific to S molecular form	Common to both forms	Total number of loci with an insertion	M molecular form	S molecular form
*Aara8*	4	3	3	110	6.58±1.71	6.69±1.43
*Ozymandias*	5	2	12	245	19.41±5.78	12.99±3.73
*Crusoe*	4	2	7	169	10.33±3.01	11.67±3.84

**Figure 7:**
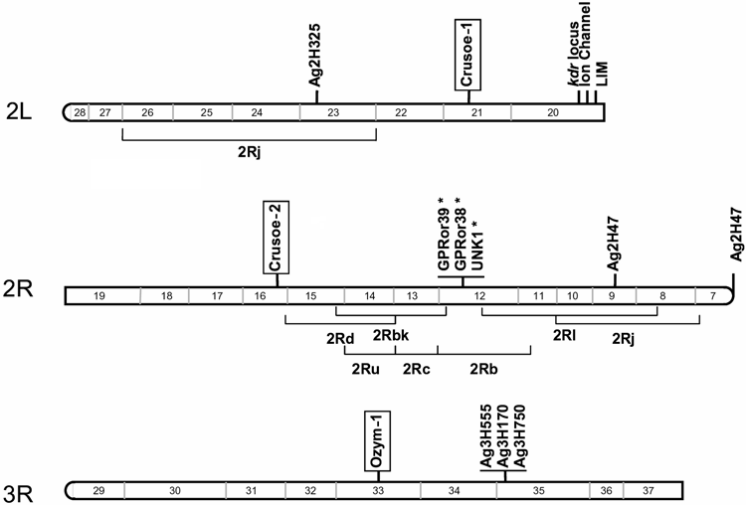
Position on chromosomes 2 and 3 of the three TE insertions specific to the M molecular form (in box). The loci Ag2H325, Ag2H417, Ag2H769, Ag3H555, Ag3H170, Ag3H750 from [*8*], *kdr* from [*4*], and Ion channel and LIM from [*17*], have been shown to differentiate the two M and S forms in previous studies. The chromosomal inversions of the *An. gambiae* genome are indicated below the chromosome arms. The GPRor39, GPRor38, and UNK1 loci, indicated by asterisks, have been shown to discriminate between the two forms only in Cameroon [*16*].

To localize the specific TE insertions on the chromosome arms of *An. gambiae*, we extracted the corresponding ^33^P labeled bands from polyacrylamide gels, and sequenced the DNA to make sure that the bands corresponded to the expected TEs and to obtain the sequences flanking the TEs. Among the 20 specific insertion sites that were attempted to be sequenced (13 on M and 7 on S), 7 were not isolated, 7 were isolated but were found to be integrated within repeated sequences or transposable elements and could not be localized, 3 were located in the “unannotated” chromosome. This suggests that some of these insertions were embedded within the heterochromatin or were inserted within nests of TEs, which could themselves be heterochromatic. The localization of some of the TEs specific to one of the molecular forms within the heterochromatin, raises the important possibility that drastic differences in the composition of heterochromatin may exist between populations, and the question of the influence of heterochromatin on genetic differentiation and speciation processes, once again highlighting the need for more intensive research on this particular genomic region [23]. Three of the specific insertions were however unambiguously localized on chromosomes. They consisted of two *Crusoe* (*Crusoe*-1, *Crusoe*-2) and one *Ozymandias* (*Ozym*-1) insertions specific to the populations of the M molecular form. These insertions were localized in the division 21 of the 2L chromosome (outside the known inversions), and division 16 of the 2R chromosome (outside the 2Rd inversion, at 800 kb from the inversion breakpoint) for *Crusoe*, and division 33 of the 3R chromosome for *Ozymandias*. These locations are outside the genomic regions previously identified as being genetically differentiated in the M and S forms [4], [8], [9], [16], 17, 19 (see Fig. 7), suggesting either that the chromosomal regions involved in this differentiation are more extensive than expected, or that there are additional differentiated regions interspersed with undifferentiated regions. More detailed analyses of these regions are necessary. It has been shown that differential population adaptation can be determined from a subset of genes while gene flow still exists between the species under speciation [24], [25]. The “islands of speciation” that define the M and S forms may thus be extending gradually, reducing gene flow and fixing some TE insertions close to the selected islands. Because the three localized insertions were outside the known inversions and not in the “islands of speciation”, these sites could result simply from genetic drift that has occurred after the separation of the M and S forms. If so, the fixed sites and the sites at high frequency would correspond to the sites of high occupancy frequency in the original founders, and the polymorphic insertion sites (sites with low occupancy frequency) would correspond to more recent transposition events, as observed in colonizing species [26]. This kind of insertion site frequency pattern is compatible with the idea that *An. gambiae* has speciated or differentiated relatively recently [27]. According to the hypothesis of founder effects, the presence of fixed TE insertion sites in each molecular form could suggest that gene flow is more restricted than it has usually been thought to be, which would be consistent with the virtual absence of hybrids in nature [8, although an unusual frequency of hybrids was found in a population from Guinea Bissau; J. Pinto, personal communication]. However, among the 6 fixed sites that we sequenced, only one (*Crusoe*-1) was localized on the chromosome arms, the others were either on the unknown chromosome, or clearly embedded within heterochromatin or other transposable elements. In addition, *Crusoe*-1, which is fixed in the M form, reaches a frequency of 0.35 in the S form, suggesting it had been a site of high frequency in the initial population from which the M and S forms both derive. The two other localized sites, *Ozym*-1 and *Crusoe*-2, which were outside the known inversions and the “islands of speciation”, were present at an intermediate frequency in the M populations (Fig 6), but at very low frequency in the S form. All these data are in agreement with founder events (26, 28) and then global expansion in Africa.

The wide distribution of *An. gambiae* suggests the possibility of population adaptations to local climatic conditions, resulting in local differentiation between populations of a same molecular form, as has indeed been observed for M populations in Cameroon and Mali, in addition to the M and S molecular form differentiation [29]. Both the M and S forms exist in Western Africa, while only the S form has been found in Eastern Africa, which implies that the S form has greater climatic adaptability or migratory capacities than the M form. Although these two forms may coexist in the same area, they appear to be in the process of incipient speciation throughout Africa. No differences have been observed in *Plasmodium* infection rates between sympatric M and S forms in Cameroon [6]. Therefore, any attempt to construct a genetically-modified, *Plasmodium*-resistant mosquito, with the intention of replacing natural, infected populations, or any other strategy of controlling *An. gambiae*, will have to take this incipient speciation between the M and S molecular forms of the mosquito into account.

## Materials and Methods

### Mosquitoes

A total of 446 *An. gambiae* mosquitoes (257 M and 189 S) were sampled from 21 sites in Africa: Ladji (6°21′ N, 2°27′ E), Lema (7°46′ N, 2°14′ E) in Benin, Vallée du Kou (VK7, 11°24′ N, 4°24′ W), Soum (12°35′ N, 2°17′ E) in Burkina Faso, Simbok (3°49′ N, 11°28′ E), Ipono (2°22′ N, 9°49′ E) in Cameroon, Bonia (10°52′ N, 1°07′ W), Mampong (5°24′ N, 0°36′ W), Odumasy (5°53′ N, 0°01′ W) in Ghana, Azureti (5°12′ N, 3°46′ W), M'be (7°14′ N, 5°1′ W), Niamoue (5°52′ N, 4°49′ W), Nieky (5°24′ N, 4°16′ W), Yaokoffikro (7°11′ N, 5°1′ W) in Ivory Coast, Banizoumbou (13°32′ N, 2°40′ E), Kosseye (13°31′ N, 2°1′ E), Zindarou (13°26′ N, 2°55′ E) in Niger, Beforona (18°58′ S, 48°15′ E) in Madagascar, Bankoumana (12°50′ N, 5°47′ W) in Mali, Furvela (23°43′ S, 35°18′ E) in Mozambique, and Zenet (5°16′ S, 38°36′ E) in Tanzania. The species and molecular form of the specimens were identified using Fanello *et al.*'s protocol [30].

### Transposon Display

Total genomic DNA was isolated from individual mosquitoes using a standard phenol-chloroform extraction procedure after proteinase K digestion. The Transposon Display was performed using a modified version of the protocol used by Zampicinini et al. [31], as follows: 50 to 100 ng of genomic DNA was digested with 10 units of *Hha*I for 6 hours at 37°C; during the first round of amplification, 3 mM of MgCl_2_ and 0.625 Units of Taq Polymerase were used; during the second amplification run, 0.2 µM of adaptor primer, 0.05 µM of nested TE-specific primer with HEX fluorescent labeling, 2.5 mM of MgCl_2_ and 0.625 Units of Taq Polymerase were used. The last steps of the nested-amplification cycles lasted 45 sec, instead of 1 min. The sequences of adaptors and primers are shown in Table S2. Negative controls were performed using the adaptor-primer or the element specific-primer alone.

The PCR products were diluted 5-fold, and 1 µl of the dilution was loaded onto a MegaBace 1000 capillary sequencer (Amersham BioSciences) with an ET900-ROX standard size marker (Amersham BioSciences). Raw data were analyzed by GeneticProfiler software (Amersham BioSciences). To confirm whether the amplified DNAs were identical to the expected TE product, 6–8 fragments were cloned using the Topo TA cloning kit (Invitrogen), following the Manufacturer's instructions, and sent to GenoScreen for sequencing. All analyzed fragments corresponded to the expected TE.

### Data analysis

Each band on the capillary gels was automatically ascribed a molecular weight according to the DNA ladder, which was loaded on each capillary. We assumed that the DNA bands with the same molecular weight shared the same TE insertion. The individual TE insertion patterns obtained from the Transposon Display were thus recorded as a binary matrix of 0 and 1 denoting the absence or presence of a given peak on the capillary gel, respectively. The between-population genetic divergence, Φst, was calculated for each pair of population samples for the three transposable elements considered separately. This Φst, which allows for the dominant nature of TE, is an analogue of the fixation index of inter-population differentiation, F_ST_ [22], [32]. Because the inter-population index values calculated from samples consisting of less than 5 individuals were not reliable, these values were not included in the calculation of the mean Φst values between populations. Graphical representations of the proximities between individuals were obtained using a Principal Coordinate Analysis (PCoA), using the R package ade4 [33]. All individuals were included in these analyses, because those from small samples were not expected to bias the results, as they were not assigned *a priori* to any specific population. For each population, we then drew the ellipses centered on the gravity center of each scatterplot, with the size of the two first axes equal to 1.5 times the standard deviation of the coordinates of the projections on the axes. MANOVA between molecular forms was performed using JMP Version 7 software (SAS Institute Inc.), and the variance components were tested for significance by nonparametric randomization tests with the null hypothesis of no population structure.

The detection of population differentiation by the PCoA is based on the sites that are either fixed or at high frequency in a form and not in the other. Hence sites with very high insertion polymorphism play no role in the differentiation.

### Identification of transposable element insertion sites

Fragments obtained from the Transposon Display were separated on a 6% denaturing polyacrylamide gel. Samples were diluted with one volume of loading dye (95% formamide, 0.05% xylene cyanol FF, and 0.05% bromophenol blue), heat denatured at 95°C for 5 min, and immediately cooled on ice. Polyacrymamide gel was pre-run at 75 W for 30 min. Six µl of each sample were run at 75 W for 4 h in 1xTBE. We used radioactive ^33^P labeling; the gel was transferred to Whatman 3 MM paper, and vacuum dried at 65°C for 1 h; dried gels were exposed to X-ray films overnight or for 48 h, depending on the signal intensity [34]. The fragments of interest were cut from the gels, the DNA was eluted from the bands at 100°C for 15 min and resuspended in 150 µl of sterile water. The fragments were amplified according to the second amplification run of the Transposon Display protocol, and cloned using the Topo TA cloning kit (Invitrogen). About 5 clones for each fragment were sequenced by GenoScreen. The genomic localizations of the sequenced DNAs were determined by interrogation of the *Anopheles gambiae* genome database (Ensembl AgamP3 assembly, release 46.3i). Among the 14 sequenced fragments, only three presented a flanking sequence localized in only one site on the chromosome arm. These three fragments corresponded to two insertions of the DNA transposon *Crusoe* and to one insertion of the LTR retrotransposon *Ozymandias*. Their specificity to the M form was confirmed by PCR. Amplifications were performed following the second amplification run of Transposon Display, using primers *Crusoe*-1F 5′-CCTATTGATTTGTCCGACACTG-3′, *Crusoe*-1R 5′-TCACTTCACGTTCGAAACAG-3′, *Crusoe*-2F 5′-CCTATTGATTTGTCCGACACTG-3′, *Crusoe*-2R 5′- TTTACCTGGC TTTTGGCAAT-3′ and *Ozym*-1F 5′-TGCTATAAGCAATCCACCACA-3′, *Ozym*-1R 5′-CTCAAAGTGTGCTTCCTCACC-3′ for the *Crusoe*-1, *Crusoe*-2 and *Ozym*-1 insertions, respectively.

## Supporting Information

Table S1 (1.58 MB DOC)

Table S2 (0.03 MB DOC)
