# Supplementary material for: High Genetic Differentiation between the M and S Molecular Forms of Anopheles gambiae in Africa
Source: PLoS One. 2008 Apr 16;3(4):e1968. doi: 10.1371/journal.pone.0001968 (PMC2278371; doi:10.1371/journal.pone.0001968)
Supplement: Table S1 — (1.58 MB DOC) [file pone.0001968.s001.doc]

**Table S1.** **Pairwise estimates of between-population genetic divergence (Φst) for *Aara8* (A), *Ozymandias* (B), and *Crusoe* (C)** **(data in bold).**

The associated P-values are shown under the diagonal (data in italic). Abbreviations for the geographic locations are as in Fig. 1. The population samples containing fewer than 5 individuals (see Fig. 1) were not included in the table.

**A**

**B**

**C**
