# Supplementary material for: High Genetic Differentiation between the M and S Molecular Forms of Anopheles gambiae in Africa
Source: PLoS One. 2008 Apr 16;3(4):e1968. doi: 10.1371/journal.pone.0001968 (PMC2278371; doi:10.1371/journal.pone.0001968)
Supplement: Table S2 — (0.03 MB DOC) [file pone.0001968.s002.doc]

**Table S2.** **Sequences of adaptors and primers used in the Transposon Display technique.**

| **Adaptors and primers** | **Sequence** | **T_M_ (°C)** | **Annealing temperature used for PCRs (°C)** | | **Length of sequence* (bp)** |
| --- | --- | --- | --- | --- | --- |
| **Adaptors**  AdaptorHhaI+ | 5’ AAC AGC TGG ACG ATG AGT CCT GAG ATA CG 3’ |  |  |  | |
| AdaptorHhaI- | 5’ TAT CTC AGG AGT GTA 3’ |  |  |  | |
| **Primers**  PrimerHhaI+ | 5’ AAC AGC TGG ACG ATG AGT 3'’ |  |  |  | |
| Aara8Ext- | 5’ AAC ACT TCA CTG TGA TAC AG 3’ | 56 | 56 | 211 | |
| Aara8Nested- | 5’ GCA TTG AAC GGC TAA AAG 3’ | 52 | 56 | 151 | |
| OzymandiasExt- | 5’ CGA TGT AAA CCG TTC TAA G 3’ | 54 | 56 | 148 | |
| OzymandiasNested- | 5’ TTC GAT ATT CAG TGT AAC CAC 3’ | 58 | 56 | 95 | |
| CrusoeExt- | 5’ TCA ACA AAC TTT CAA AGC A 3’ | 50 | 52 | 163 | |
| CrusoeNested- | 5’ ATC CGA AAA GTG CTT TCA 3’ | 50 | 52 | 129 | |

* Length of sequence (bp) between the primer and the 5’ end of the TE. The T_M_ and annealing temperature of the primer HhaI+ depended on the transposable element concerned by the PCR.
